# Production and Purification of Pectinase from *Bacillus subtilis* 15A-B92 and Its Biotechnological Applications

**DOI:** 10.3390/molecules27134195

**Published:** 2022-06-29

**Authors:** Yahya S. Alqahtani, Sunil S. More, Keerthana R., Ibrahim Ahmed Shaikh, Anusha K. J., Veena S. More, Francois N. Niyonzima, Uday M. Muddapur, Aejaz A. Khan

**Affiliations:** 1 Department of Pharmaceutical Chemistry, College of Pharmacy, Najran University, Najran 66462, Saudi Arabia; ysalmuneef@nu.edu.sa; 2 School of Basic and Applied Sciences, Dayananda Sagar University, Bangalore 560111, India; keerthanaraju10@gmail.com (K.R.); anushak28@gmail.com (A.K.J.); 3 Department of Pharmacology, College of Pharmacy, Najran University, Najran 66462, Saudi Arabia; i.ibrahimshaikh09@gmail.com; 4 Department of Biotechnology, Sapthagiri College of Engineering, Bangalore 560157, India; veenasmore@gmail.com; 5 Department of Math, Science and PE, CE, University of Rwanda, Rwamagana P.O. Box 55, Rwanda; niyofra@yahoo.com; 6 Department of Biotechnology, KLE Technological University, Hubballi 590031, India; muddapur@kletech.ac.in; 7 Department of General Science, Ibn Sina National College for Medical Studies, Jeddah 21418, Saudi Arabia; aeju_kh@yahoo.com

**Keywords:** screening, citrus pectin, pectinase, *Bacillus subtilis* 15A-B92, purification, juices clarification

## Abstract

Enzymes that degrade pectin are called pectinases. Pectinases of microbial origin are used in juice clarification as the process is cost-effective. This study screened a pectinase-producing bacterium isolated from soil and identified as *Bacillus subtilis* 15A B-92 based on the 16S rRNA molecular technique. The purified pectinase from the isolate showed 99.6 U/mg specific activity and 11.6-fold purity. The molecular weight of the purified bacterial pectinase was 14.41 ± 1 kD. Optimum pectinase activity was found at pH 4.5 and 50 °C, and the enzyme was 100% stable for 3.5 h in these conditions. No enzymatic inhibition or activation effect was seen with Fe^2+^, Ca^2+^, or Mg^2+^. However, a slight inhibition was seen with Cu^2+^, Mn^2+^, and Zn^2+^. Tween 20 and 80 slightly inhibited the pectinase, whereas iodoacetic acid (IAA), ethylenediaminetetraacetate (EDTA), urea, and sodium dodecyl sulfate (SDS) showed potent inhibition. The bacterial pectinase degraded citrus pectin (100%); however, it was inactive in the presence of galactose. With citrus pectin as the substrate, the Km and Vmax were calculated as 1.72 mg/mL and 1609 U/g, respectively. The high affinity of pectinase for its substrate makes the process cost-effective when utilized in food industries. The obtained pectinase was able to clarify orange and apple juices, justifying its application in the food industry.

## 1. Introduction

Pectinases hydrolyze pectic compounds linked by α-1,4-glycosidic bonds and esterified with methyl groups. Pectins, protopectins, and pectinic acids or polygalacturonic acids are the main constituents of heterogenic pectic substances. Based on their mode of action, pectinases are classified as polygalacturonases that hydrolyze unesterified polygalacturonic acid substances, pectin esterases, pectin lyases that de-esterify pectin into pectate and methanol, and pectate lyases or polymethylgalacturonases that catalyze β–elimination, forming galacturonides. Polygalacturonases, pectin lyases and pectate lyases have been found to show the highest activity among the pectinases. A homology at the sequence level between these pectinolytic enzymes has also been observed. The main natural sources of pectinases are bacteria, fungi, and plants [1,2].

The main producers of pectinases are *Bacillus*, *Aspergillus*, *Saccharomyces* sp., etc. To produce pectinases in significant amounts, submerged and solid-state fermentations are utilized. Pectinases play a significant role in the food industry, oil extraction, the textile industry, coffee and tea fermentation, etc. Acidic pectinases clarify juices at significant levels. They increase the yield of juice by pulp liquefaction. A worldwide share of 25% was reported for pectinolytic enzymes among food enzymes [3,4]. Some studies have been carried out to isolate bacteria producing pectinases with desirable properties [5,6,7,8,9,10,11,12,13,14,15,16,17,18]. The research on bacteria that produce higher pectinase levels is a continuous exercise. The objective of the study was therefore to overproduce bacterial pectinase and to characterize and apply it to juice clarification.

## 2. Materials and Methods

### 2.1. Screening and Identification of Pectinase-Producing Bacterium

Bacteria were screened using 3 different sources, viz. fruits, soil, and vegetables. Fresh fruits and vegetables were collected at a nearby market and were ground, spoiled, and dried in sunlight. Soil samples were collected in horticulture fields. The soil samples were sterilized and exposed to sunlight. A total of 1 g of each sample was mixed with 100 mL normal saline, and serially diluted. A volume of 0.1 mL of each diluted sample was put in LB agar plates at pH 6.5. The incubation was performed at 37 °C for 48 h. The screening medium was composed of 1% citrus pectin, 0.14% (NH_4_)_2_SO_4_, 0.6% K_2_HPO_4_, 0.20% KH_2_PO_4_, 0.01% MgSO_4,_ and agar. Pectinase production was indicated by a clear halo zone around the colonies after the Petri plates were flooded with iodine solution. Culture aspects, Gram and endospore staining, and biochemical tests were used to identify the bacterial isolates showing the highest pectinase activity. The identification of species names was conducted by Barcode Biosciences (Bangalore, India).

### 2.2. Production of Bacterial Pectinase

The production medium at pH 6.5 was the same as the one used for the isolation and screening but with no agar on it. The bacterium with the highest clear zone diameter was considered with 1% inoculum. The inoculated fermentation medium was incubated at 37 °C on a shaking incubator for 48 h. After incubation, the sample was centrifuged at 10,000 rpm for 5 min at 4 °C. The supernatant was collected and used as a crude enzyme. The optimization of the fermentation medium was also carried out with different N sources (tryptone, potassium nitrate, yeast extract), temperatures (30, 40, and 50 °C), pH (3.5, 4.5. 5.5, 6.5, and 7.5), and incubation times (24, 36, and 48 h), changing one factor each time and maintaining the other factors unchanged.

### 2.3. Measuring Enzyme Activity and Protein Content

The 3,5-Dinitrosalicylic acid [19] and Lowry et al. [20] methods were followed for pectinase activity and protein content determinations at 540 and 660 nm, respectively, with citrus pectin and bovine serum albumin as standards. The enzyme activity (U) was defined as the amount of enzyme that produced 1 µmol of product per min per ml under the assay conditions.

### 2.4. Bacterial Pectinase Purification

Partial purification was performed by sequential ammonium sulphate saturation (0–60, 60–80, and 80–90%), dialysis, and then lyophilization. Affinity chromatography with agarose-bound lectins was utilized to completely purify the enzyme, as suggested by Spivak et al. [21]. The bound fraction with a significant amount of protein and pectinase activity was used for the pectinase characterization. Sodium dodecyl sulfate polyacrylamide gel electrophoresis (SDS PAGE) by Laemmli [22], followed by silver staining [23], was conducted for determining the homogeneity and relative molecular mass using standard molecular weight markers. The molecular mass was also re-checked by liquid chromatography–mass spectrometry (LC–MS) [24].

### 2.5. Physico-Chemical Properties of Bacterial Pectinase

The influence of temperature on pectinase activity was studied in the 0–90 °C range. The pH optimum was determined over a range of pH 3.0 to 10.0 with different buffer solutions. The stability of the pectinase at the optimum temperature and pH was also investigated at different intervals of time. The effect of metal ions (Fe^2+^, Ca^2+^, Cu^2+^, Mn^2+^, Zn^2+^, and Mg^2+^), surfactants (Tween 20 and Tween 80), and specific reagents (β-mercaptoethanol, EDTA, SDS, urea, and IAA) on the activity of pectinase was investigated by adding different known concentrations of cations to the mixture of pectinase and its substrate. Citrus pectin, apple pectin, xylan, and galactose were the substrates used to study the broad substrate specificity of the purified enzyme. Km and Vmax were quantified with a double reciprocal plot using citrus pectin as the best substrate. In all cases, the enzyme assay was carried out at the optimum pH and optimal temperatures, and the activity of the pectinase was quantified as described earlier.

### 2.6. Application of Bacterial Pectinase in Apple and Orange Juice Clarification

The Kumar and Suneetha [25] method was followed with modifications: 1 kg of apples were washed, cut into small cubes, and crushed using a juice processor. The obtained juice was pasteurized in a water bath for 5 min at 85 °C. A total of 50 mL of each cooled apple juice was mixed with different pectinase concentrations (0, 0.5, 1, 2, and 5%). The incubation was then performed at 50 °C for 1 h in a water bath. The reaction was stopped by heating the mixture at 90 °C for 5 min. The pH, viscosity, and turbidity were checked with a pH meter, viscometer, and turbidimeter, respectively. The juice clarity was quantified at 660 nm. The experiment was repeated with oranges (instead of apples) in the same conditions.

### 2.7. Statistical Analysis

The data on pectinase activity was obtained in triplicate. Analysis of variance (ANOVA) and Duncan’s multiple range tests (DMRT) were utilized to analyze the data, with the help of SPSS, at a significance level of 5%.

## 3. Results and Discussion

### 3.1. Isolation and Identification of Pectinase-Producing Bacteria

**Figure 1:**
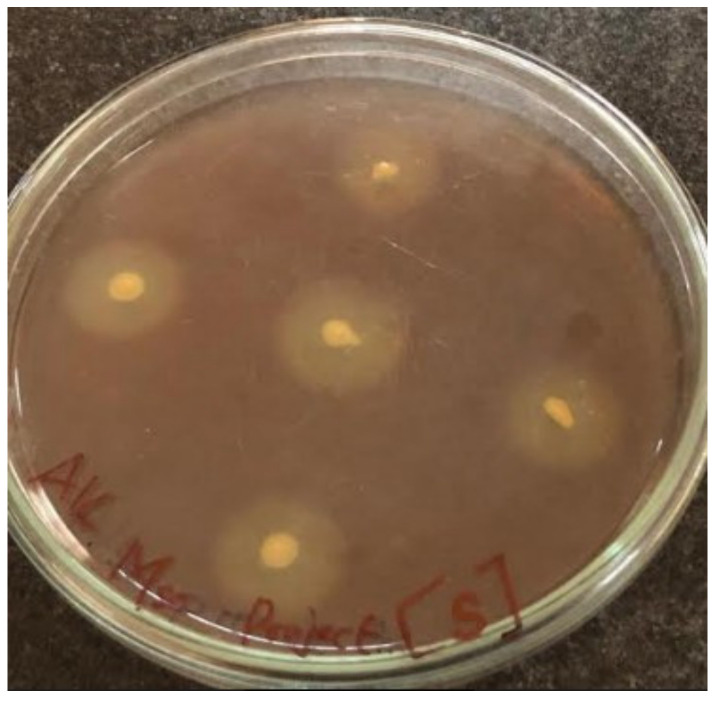
Bacterial isolate showing zone of hydrolysis after iodine solution application.

**Figure 2:**
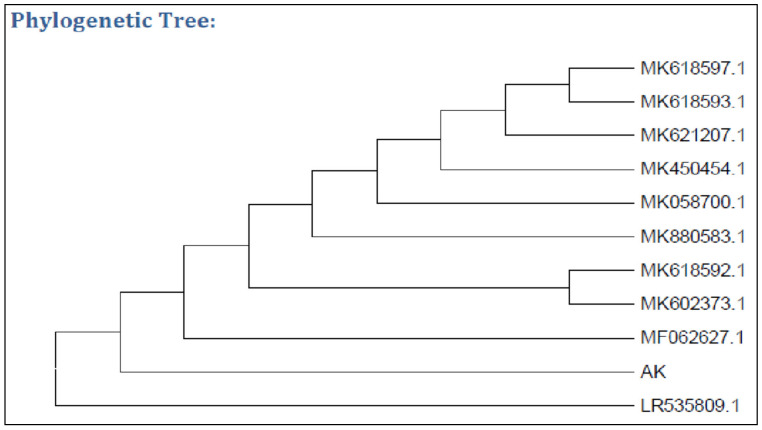
Phylogenetic tree showing the position of isolated bacterium (AK).

Screenings for pectinase-producing bacteria were carried out with fruit, soil, and vegetable samples using the plate culture method. Among the 20 bacterial isolates, 9 isolates displayed clear zones which indicated the existence of pectinase activity. After iodine staining, the bacterium with the highest pectin hydrolysis clear zone (Figure 1) was subjected to identification. The bacterium was found to be *Bacillus* based on its morphological and microscopic aspects, and *Bacillus subtilis* based on the Gram staining and biochemical characteristics from Bergey’s manual of determinative bacteriology [26]. The bacterium was further confirmed by molecular techniques (using 16S rRNA) as being *Bacillus subtilis* 15A B-92 using the Basic Local Alignment Search Tool (BLAST; Figure 2). Bacteria-producing pectinases were also reported [5], with citrus pectin as an inducer. Various fungi-producing pectinases were also isolated [7,8,27].

### 3.2. Production of Bacterial Pectinase

Bacterial pectinase was produced extracellularly from the isolated *Bacillus*. The citrus pectin was used as an inducer. The culture conditions were optimized in order to produce bacterial pectinase in significant amounts. The organic (tryptone and yeast extract) and inorganic (potassium nitrate) nitrogen sources did not increase pectinase activity. However, the submerged fermentation (with pH 4.5) at 40 °C for 36 h resulted in maximal pectinase activity. Similarly, 36 h and 37 °C were the optimum conditions for pectinase production by *Bacillus* sp. DT-7, but under solid state fermentation [5]. Citrus pectin was also the best carbon source and inducer for microbial pectinase production under submerged fermentation [27]. Similarly, yeast extract was the best organic nitrogen source for pectinase production by *Bacillus subtilis* SAV-21 [8]. A higher incubation period of 96 h (at 30 °C) was seen for pectinase secretion by *Aspergillus niger* [9]; 40 °C was also optimum pectinase activity for pectinase of *A. niger* URM 46,457.5 [28]. A short incubation time at the desired temperature makes the production cost-effective.

### 3.3. Pectinase Purification

The crude enzyme preparation was purified by conventional methods. The purified pectinase showed 99.6 units/mg (U/mg) of specific activity with 11.6-fold higher purity and a final yield of 18.8% (Table 1). Similarly, a polygalacturonase-obtained *Bacillus subtilis* C4 was partially purified by ammonium sulphate salt and totally purified by affinity chromatography, with 13,789.11 U/mg specific activity and 15-fold higher purity [29]. *Penicillium viridicatum* Rfc3 also produced pectin lyase and polygalacturonase with specific activities of 2000 and 30 U/g, respectively [10].

**Figure 3:**
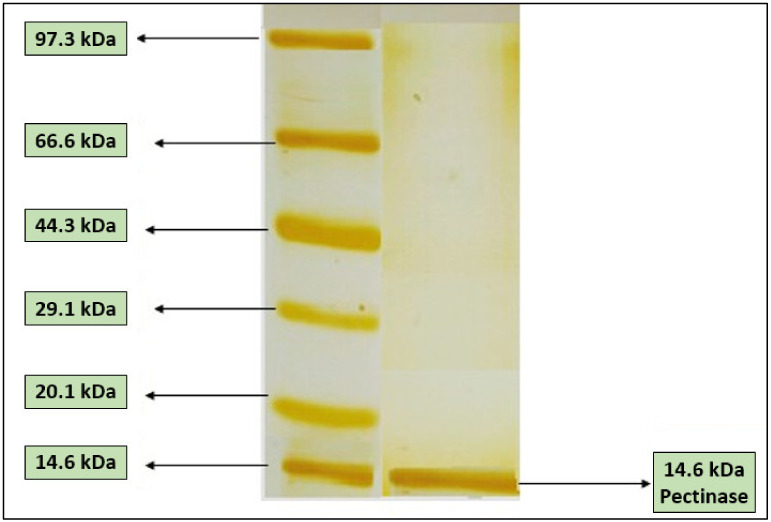
SDS PAGE pattern of purified pectinase purified from *Bacillus subtilis* 15A-B92.

**Figure 4:**
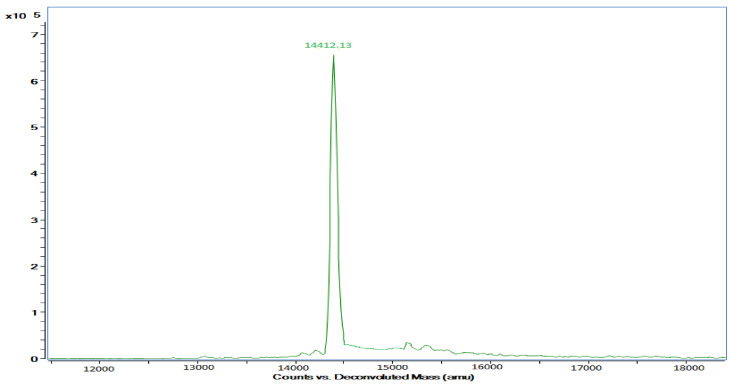
Deconvoluted mass spectrum of bacterial pectinase from *Bacillus subtilis* 15A-B92.

After SDS, native PAGEs, and silver staining, the pectinase was found to be a monomer with a molecular weight of 14.41 ± 1 kDa, as estimated using standard molecular weight markers (Figure 3) and LC–MS (Figure 4). Similarly, a similar molecular mass of 14.13 kDa was reported for a fungal pectinase of *Aspergillus repens* after ammonium sulphate partial purification, molecular exclusion, and ion-exchange chromatography purification procedures [11]. A low molecular weight of 6.5 kDa was reported for the pectinase purified from *Bacillus coagulans* after SDS-PAGE. The molecular mass of the bacterial polygalacturonase purified from *Bacillus subtilis* C4 was higher and was in the 43–66 kDa range [29].

### 3.4. Physico-Chemical Properties of Bacterial Pectinase

#### 3.4.1. Effect of Temperature on the Activity and Stability of Pectinase from *Bacillus subtilis* 15A-B92

A change in enzyme activity at different temperatures was observed owing to changes in the native structure at the active site. Changes in the native structure affect the affinity of binding of the substrate, leading to an increase or decrease in activity. This alters the enzyme’s functional shape [30]. The effect of temperature on the purified pectinase was examined in the 0–90 °C range; 50 °C was recorded as the maximum temperature with maximum pectinase activity. Pectinase activity decreased after the maximum pectinase activity; this may be a result of pectinase thermal denaturation owing to non-covalent linkage disruption. A similar decrease in pectinase activity was reported by Phutela et al. [6] for pectinase from *A. fumigates* and by Alana et al. [31] for pectinase purified from *Penicillium italicum*. Penctinase stability was checked for at different intervals and 100 and 60% residual activity were recorded after 3.5 and 24 h, respectively. Nadaroglu et al. [12] purified a pectinase that was only stable for 1 h, with a loss of enzyme activity seen after that period. Arotupin et al. [11] found a maximum temperature of 30 °C for pectinase from *Aspergillus repens*. Polygalacturonase and pectin lyase purified from *Penicillium viridicatum* Rfc3 were reported at 40 and 35 °C, respectively; they were stable for only 1 h at their optimum temperatures [10]. Therefore, the pectinase purified from the present *Bacillus subtilis* 15A-B92 appears to have a higher maximum stability than other reported pectinases.

#### 3.4.2. Effect of pH on the Activity and Stability of Pectinase from *Bacillus subtilis* 15A-B92

Changes in enzyme activity at different pH values is observed due to changes in the ionization of the amino acids residues in the active site. A change in the ionization state affects the affinity of binding of the substrate, leading to an increase or decrease in activity [30]. The influence of pH on bacterial pectinase was rechecked in the three to nine range with 0.5 intervals. Maximum pectinase activity was noticed at pH 4.5. The pectinase was 100% stable for 3.5 h and a retention of enzyme activity of 70% was seen after overnight incubation at 50 °C. A higher pH of 6.5 was observed for pectinase obtained from *Aspergillus repens* [11]. Similarly, a maximum pH of 5.0 was seen for polygalacturonase from *Penicillium viridicatum* Rfc3; it was also stable at neutral pH for only 1 h [10]. A higher pH stability was thus seen for the present pectinase compared to other pectinases. Thus, the pectinase of the present isolated bacterium can be applied to various industries owing to its stability over a range of pH values.

#### 3.4.3. Effect of Divalent Cations on the Activity of Pectinase from *Bacillus subtilis* 15A-B92

Metal ions may have various effects on pectinase active site activity. Fe^2+^, Ca^2+^, and Mg^2+^ were found to have no influence on enzyme activity, whereas Cu^2+^, Mn^2+^, and Zn^2+^ showed slight inhibition. Arotupin et al. [11] produced pectin methylesterase (PME) from *Aspergillus repens* and the enzyme was activated by Na^+^, Ca^2+^, K^+^, Mg^2+^, and Zn^2+^. However, inhibition was seen with Hg^2+^. Similarly, Mg^2+^ and Zn^2+^ did not affect pectin lyase activity [31]. Divalent cations, especially Ca^2+^ and Mg^2+^, are known to maintain the enzyme-like pectinase active site [32]. This discrepancy in the actions of metal cations on pectinase activity may be attributed to different and various functional groups in the active site.

#### 3.4.4. Effect of Inhibitors and Surfactants on Bacterial Pectinase

The surfactants used were Tween 20 and 80 and were found to slightly inhibit the enzyme. Contrastingly, Li et al. [33] purified a polygalacturonase that was stimulated by Tween-20 and Tween-80. The same stimulatory effect of pectinase was also reported by Zu-ming et al. [13]. The stimulatory effect of surfactants may improve interactions between the pectinase active site and the substrate. In the inhibition study, a slight inhibition was seen for β-mercapethanol. However, a strong inhibition was seen with IAA, EDTA, urea, and SDS for isolated bacterial pectinase. Similarly, EDTA and IAA strongly inhibited the pectinase obtained from *Aspergillus repens* [11]. SDS also inhibited a pectinase isolated by Amid et al. [14]. Thus, pectinase stability and activity, like other enzymes, is affected by pH, temperature, activators, and/or inhibitors. Since the pectinase in the present study was almost stable with various inhibitors and surfactants, it could be used in various industries for pectin substrate degradation.

#### 3.4.5. Substrate Specificity and Kinetic Determinations of Bacterial Pectinase

The degradation of different pectic compounds such as citrus pectin, galactose, xylan, and apple pectin by bacterial pectinase was investigated. Maximum hydrolysis was seen for citrus pectin. 50% degradation was seen for apple pectin and xylan. However, 0% degradation was noted for galactose. Similarly, the maximum pectinase activity was noticed during citrus pectin degradation by the enzyme of *Acrophialophora nainiana* [15]. Kinetic parameters help in knowing the affinity and catalytic efficiency of the purified pectinase for its substrate. With citrus pectin as the substrate, the Km was 1.72 mg/mL and the Vmax was 1609 U/g. A similar Km of 1.88 was seen for pectinase obtained from *Bacillus subtilis* Btk 27 [16]. A low Km value of 1 mg/mL was also seen for pectinase from *P. chrysogenum*, while a higher Km value of 4.22 mg/mL was found for pectinase from *Acrophialophora nainiana* [15]. The low affinity recorded for pectinic substrates obtained from *Bacillus* species highlights the use of pectinase in industrial factories. The high affinity of pectinase for its substrate may render the industrial process cost-effective.

### 3.5. Application of Bacterial Pectinase in Apple and Orange Juice Clarification

**Figure 5:**
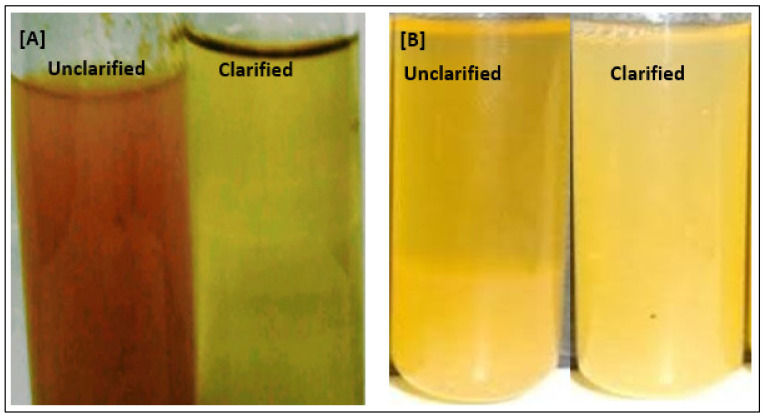
Juice clarification. (**A**): unclarified and clarified apple juice. (**B**): unclarified and clarified orange juice.

Apple and orange juice clarification was investigated using the crude pectinase of *Bacillus subtilis*. The results revealed that there was a decrease in their turbidity and that their pH was not altered (Figure 5). A 50 and 59% reduction in viscosity was observed for the orange and apple fruits, respectively, compared to untreated juices. The pectinase from *Aspergillus awamori* was able to clarify orange juice and an improvement in clarity of 95% was observed [34]. Kareem and Adebowale [27] reported orange juice clarification by turbidity and viscosity reductions when pectinase from *Rhizopus oryzae* was utilized. The fungal pectinase obtained from *Aspergillus niger* [17], *Aspergillus awamori* Nakazawa MTCC 6652 [35], and *Penicillium oxalium* F67 [18] improved the degree of clarification and decreased the viscosity and turbidity to acceptable levels for apple juice. The mechanism involved in juice clarification is that the microbial pectinase removes methyl groups from pectin backbone. Then, the negatively charged regions of the pectin get complexed with Ca^2+^ to form Ca^2+^ pectate gels, aiding in juice clarification [25]. As the pectinase obtained from *Bacillus subtilis* 15A B-92 had superior clarity, turbidity, and viscosity reduction compared to other microorganisms, it may be a good choice in industrial juice clarification.

## 4. Conclusions

In the present investigation, the pectinase of *Bacillus subtilis* 15A-B92 was produced, characterized, and evaluated for its applicability. The pectinase was found to be important for juice industries owing to its desirable acidic pH, temperature, surfactant stability, and very low Km. The present pectinase could thus be a promising choice for juice clarification.

## Figures and Tables

**Table 1:** Summary of the purification of *Bacillus subtilis* 15A-B92 pectinase.

Purification Step	Total Activity (U)	Total Protein (mg)	Specific Activity (U/mg)	Purification Fold-Change	Purification Yield
Crude enzyme	4352.0	506.0	8.6	1	100
Ammonium sulfate precipitation	1602.5	51.9	30.9	3.6	36.8
Affinity chromatography	816.9	8.2	99.6	11.6	18.7

## Data Availability

Not applicable.

## Sample Availability

Samples of the compounds are available from the corresponding author.
